# A Biomimetic Solvatochromic Chip With Optimized Chemical Diversity for On‐Site Liquid Identification

**DOI:** 10.1002/advs.78158

**Published:** 2026-10-08

**Authors:** Jintao Zhang, Hao Ouyang, Yin Cheng, Nuoye Xiong, Xunchi Ma, Xueling Li, Guangming Zhu, Liang Zhang, Jing Sun, Ranran Wang

**Affiliations:** ^1^ The State Key Laboratory of High Performance Ceramics Shanghai Institute of Ceramics Chinese Academy of Sciences Shanghai China; ^2^ Center of Materials Science and Optoelectronics Engineering University of Chinese Academy of Sciences Beijing China; ^3^ School of Computer Science and Technology Xidian University Xi'an China; ^4^ School of Chemistry and Materials Science Hangzhou Institute for Advanced Study University of Chinese Academy of Sciences Hangzhou China

**Keywords:** biological system, chip, cloud computing, fabrication, nanotechnology, solvatochromism

## Abstract

Colorimetric sensors for instant and on‐the‐spot identification of organic liquids have been actively developed across diverse fields such as chemical hazard inspection, manufacturing process monitoring, and beverage safety. Multi‐target sensing scenarios often necessitate large‐scale sensor arrays, which inevitably increase the fabrication complexity, complicate visual interpretation, and exacerbate data redundancy. Here, we present a biomimetic photonic‐gustatory chip (BiPC) comprising a polyacrylate‐based solvatochromic disk (PSD) array. The elastic photonic crystal PSDs generate distinct color patterns when swelled by organic solvents. To fully unlock the sensing potential, a Hansen solubility parameter (HSP) framework was developed to assess the overall swelling cross‐reactivity toward target liquids, facilitating the design of a compact PSD array with HSP‐guided chemical diversity and maximized inter‐solvent response distinctiveness. Using intuitive pattern recognition, an HSP‐optimized four‐PSD array effectively differentiates solvent types and mixing ratios, homologues and isomers, as well as alcoholic beverages. Furthermore, we developed a smartphone application that integrated image capture with deep‐learning‐assisted cloud computing for on‐site solvent identification, achieving high accuracy (97.1% across 80 liquid classes) within 30 s. The HSP framework is generalizable to the design of compact sensor arrays for various field‐deployable liquid sensing scenarios.

## Graphical abstract

A compact biomimetic photonic‐gustatory chip uses Hansen‐solubility‐parameter‐guided solvatochromic disks to create distinct color patterns in organic liquids. An optimized four‐disk array distinguishes solvent types, ratios, homologues, isomers, and alcoholic beverages. Coupled with smartphone imaging and deep learning, it identifies 80 liquid classes with 97.1% accuracy in 30 s, enabling field‐deployable liquid sensing.

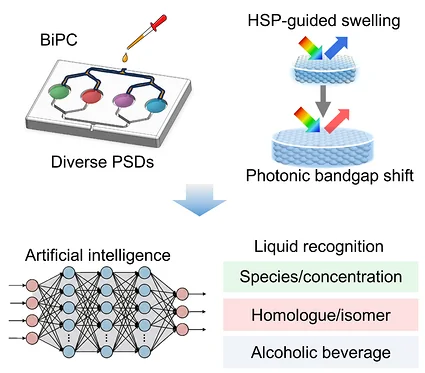

## 1 Introduction

A rapid and accurate technology for identifying organic liquids has long been sought after due to its broad significance in applications ranging from chemical hazards inspection and food and drink safety to manufacturing process monitoring [1, 2, 3, 4, 5, 6, 7]. While sophisticated analytical techniques such as chromatography and mass spectrometry offer high precision, they require expensive and bulky equipment, long operation time, and highly trained personnel [8]. Consequently, various simpler alternative approaches have been proposed. For instance, gel‐based dynamic reflection spectrum (DRS) can accurately recognize different organic solvents; however, it depends on a UV–vis spectrometer for data acquisition, and the liquid exchange process is time‐consuming (approximately 10 min) and susceptible to temperature variation [9]. Song et al. developed a transparency‐switching medium based on light scattering for liquid sensing. Although easily accessible, the limited number of indicator variables (i.e., the optical states of transparent, semitransparent, and opaque) hinders the attainment of high identification efficiency, defined as the number of detectable types per sensing element [10]. A droplet‐based triboelectric‐visual system enables deep learning‐assisted rapid identification within seconds. Nevertheless, its operation requires an electrometer and a high‐resolution industrial camera, which compromises portability for on‐site use [11].

Colorimetric sensing presents a low‐cost and user‐friendly strategy for intuitive readout and is compatible with convenient yet powerful imaging tools, such as smartphone cameras [12, 13, 14, 15]. Typically, colorimetric liquid sensors produce perceivable changes in chemical or structural colors when exposed to target liquids. Chemical approaches involve the use of chemoresponsive dyes [16, 17, 18], metal‐organic framework (MOF)‐based colorants [19, 20, 21, 22], and π‐conjugated polydiacetylenes (PDAs) and their derivatives [23, 24, 25, 26]. Despite the diversity of available chemical species, concerns regarding chemical colorimetry persist, including the complex synthesis processes, the need for large‐scale sensing arrays due to the high selectivity of individual sensors, and long detection times associated with slow reaction kinetics. In contrast, colorimetric sensors based on photonic structures have gained substantial interest owing to the combined advantages of easy fabrication, long‐term stability, environmental friendliness, and most importantly, broad tunability across the visible spectrum. Pioneering studies employing opal, inverse opal, and cholesteric structures leverage structural color changes to discern organic liquids [27, 28, 29, 30, 31], a process governed primarily by the polymer matrix swelling that regulates the photonic lattice. However, the lack of rational design in the polymer matrix diversity limits the range of detectable species, particularly for organic solvents with similar chemical structures. To date, developing a rapid, accurate, and highly efficient technology for versatile on‐site identification of organic liquids remains a formidable challenge.

Herein, we report a biomimetic photonic‐gustatory chip (BiPC) comprising a microfluidic vessel and an array of polyacrylate‐based solvatochromic disks (PSDs). The PSDs are elastic photonic crystals consisting of silica nanosphere assemblies embedded in polyacrylate matrices with varied side‐chain chemistry. Departing from the conventional large‐scale sensor array strategy, we exploit the Hansen solubility parameter (HSP) theory to assess the overall swelling cross‐reactivity toward target organic liquids, thereby unlocking the potential sensing capability of a compact PSD array. The HSP‐guided chemical diversity maximizes the inter‐solvent response distinctiveness to enhance identification efficiency. Two intuitive pattern recognition schemes, namely colorimetric cards and RGB radar fingerprints, prove effective in differentiating a wide variety of organic liquids, including organic solvent species and their mixing ratios, homologues and isomers, and the organic components of Chinese Baijiu and other liquors. Utilizing deep learning algorithms, the BiPC can recognize eight organic solvents across ten concentration gradients with a prediction accuracy of up to 99.7%, achieving a notably high identification efficiency of 20 (corresponding to 80 detectable classes using a four‐element sensor array). We further developed a smartphone‐based recognition application, which integrated mobile front‐end devices with image processing technology on the server side, establishing a robust pipeline that translates raw sensor data into high‐precision identification results through efficient cloud‐to‐edge coordination. The proposed BiPC‐based recognition platform is rapid (within 30 s), reusable (for at least 50 cycles), and thermally stable (from −20°C to 50°C), requiring no additional equipment or professional training. The BiPC technology holds considerable promise for general‐purpose liquid differentiation and can inspire the design of compact yet highly efficient colorimetric sensor devices for diverse sensing scenarios. Our work introduces three key innovations that collectively advance the state‐of‐the‐art: (i) a rational HSP‐based framework for designing compact sensor arrays with maximized cross‐reactivity, departing from the conventional trial‐and‐error approaches; (ii) the successful demonstration that a four‐element array can achieve high‐accuracy discrimination of 80 liquid classes with an identification efficiency of 20 classes per sensing element; and (iii) the integration of these elements into a practical smartphone‐based platform with cloud‐assisted deep learning for automated on‐site recognition.

## 2 Results

### 2.1 Design Concept of the BiPC‐Based Liquid Sensing System

The human gustatory system enables the perception and recognition of a multitude of liquid compounds. Remarkably, the taste bud, as the functional unit, contains a limited repertoire of taste receptors (namely, sour, salty, sweet, and bitter), yet responds to a diverse range of tastes. These taste receptor cells (TRCs) generate neuroelectric signals that are relayed to the gustatory cortex and collectively interpreted as distinct taste sensations (Figure 1a). Inspired by this human gustatory mechanism, we developed a BiPC‐based liquid sensing platform for rapid and accurate recognition of organic liquids (Figure 1b). The BiPC consists of an array of PSDs encapsulated within a customized microfluidic chip (fabrication details in Section 4 and Figure S1). The PSDs are constructed using hexagonal lattices of silica nanospheres embedded in a solvent‐free polyacrylate matrix (Figure 1c, preparation details in Section 4 and Figure S2). The acrylate groups are bound on the surface of silica nanospheres via hydrogen bonds with silanol groups, resulting in a solvation layer to be captured after photopolymerization. The solvation layer exerts interparticle repulsion to facilitate formation of a non‐close‐packed regular array of silica nanospheres. Upon solvent exposure, swelling induces a red‐shift in photonic bandgap, manifesting as a visible structural color change. By adjusting the volume fraction of silica nanospheres, PSDs derived from different polyacrylates exhibit similar bluish hues (Figure S3), with negligible variation across fabrication batches (Figure S4). Hydrogen bonding between acrylate groups and silanol groups ensures excellent structural stability (Figure S5), with color persistence for at least ten months under storage temperatures ranging from −10°C to 80°C. It is worth noting that the hydrophobic nature of the polyacrylate matrix, combined with the surface passivation of silica nanospheres via hydrogen bonding, renders the PSDs largely insensitive to ambient humidity fluctuations. This insensitivity is consistent with the HSP prediction that water lies far outside the solubility spheres of all selected polyacrylates, and is corroborated by the reproducible color responses obtained under ambient conditions across a wide temperature range. Preliminary experiments confirmed that the photonic bandgap shift is governed primarily by the lattice expansion due to polyacrylate swelling, rather than refractive index change upon solvent infiltration (Note S1, Figure S6). Building on this insight, we employed the HSP model to evaluate the cross‐reactive swelling responses toward target liquids, aiming to maximize the discrimination power of a compact PSD sensor array (Figure 1d). The equilibrium swelling of the PSDs is governed by both the polymer‐solvent compatibility (as described by HSP theory) and the network cross‐linking density. The interplay between cross‐linking density and solvent affinity provides an additional dimension for tuning the sensor array's response diversity.

**FIGURE 1:**
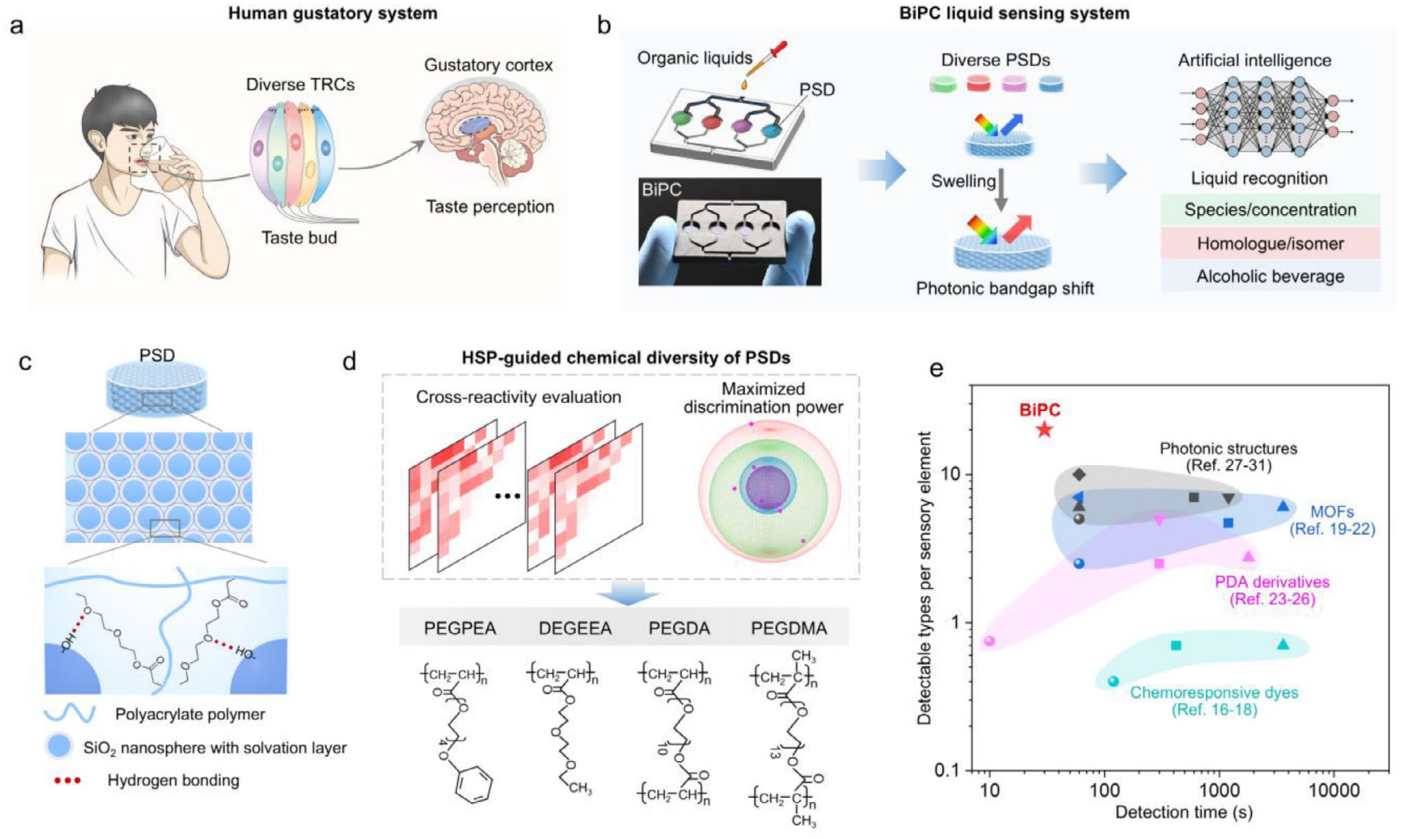
Design and working mechanism for the BiPC‐based liquid sensing system. (a) Simplified schematic of the human gustatory system, illustrating the transmission of neuroelectric signals from taste buds to the gustatory cortex. (b) Conceptual schematic of the BiPC‐based liquid sensing system. An array of PSDs with HSP‐guided chemical diversity produces distinct colorimetric response patterns, enabling deep learning‐assisted liquid identification. (c) Schematic diagram shows the non‐closely packed silica nanosphere lattice embedded in the polyacrylate matrix. The hydrogen bonding between the silanol group and the acrylate group (DEGEEA as an example here) results in the solvation layer of the silica nanosphere. (c) Conceptual schematic of HSP‐guided chemical diversity of PSDs. (d) Performance comparison of the BiPC with other colorimetric sensors in terms of detection time and the number of detectable types per sensing element.

The BiPC enables two intuitive recognition schemes, namely colorimetric cards and RGB radar fingerprints, to distinguish the organic solvent types and concentrations, homologues and isomers with similar structures and physicochemical properties, as well as organic mixtures present in Chinese baijiu and base liquors. Combined with an image‐processing deep learning algorithm, a smartphone‐based on‐site identification platform was demonstrated to achieve fast, accurate, and low‐cost organic solvent recognition. Compared to other representative solvent‐recognition mechanisms [9, 10, 11], the proposed BiPC technology excels not only in accessibility, portability, and thermal robustness, but also stands out in terms of detection speed and the number of identifiable types (detailed performance metrics in Table S1). Among colorimetric sensors [16, 17, 18, 19, 20, 21, 22, 23, 24, 25, 26, 27, 28, 29, 30, 31], the BiPC ranks at the top tier in both detection time and the number of detectable types per sensing element (Figure 1e, detailed performance metrics in Table S2). While each class of colorimetric sensors has its own merits‐for instance, chemoresponsive dyes and PDAs offer high chemical specificity, whereas MOF‐based sensors provide tailored porosity. The BiPC distinguishes itself through a combination of attributes: reversible physical swelling, fast response time, thermal robustness, and exceptionally high identification efficiency enabled by the HSP‐guided array optimization. This combination of features makes the BiPC particularly suitable for rapid, field‐deployable liquid screening applications.

### 2.2 HSP‐Guided Chemical Diversity of PSDs

Eight common industrial organic solvents with varying toxicity were selected as sensing targets (Figure 2a): dimethyl sulfoxide (DMSO), methanol (MeOH), acetone (AC), dimethylformamide (DMF), ethyl acetate (EA), dichloromethane (DCM), methylbenzene (MB), and acetonitrile (ACN). While expanding the sensor array scale can enhance identification power, it also increases device size, fabrication complexity, and data redundancy. To minimize the array while maintaining high accuracy, we sought to maximize differential swelling responses among PSDs toward target liquids. HSP theory has been proven a reliable tool to assess and predict the solubility/swelling trends of polymers in solvents [32, 33, 34, 35]. To demonstrate HSP‐guided optimization, we selected four polyacrylate monomers from six family members for constructing a compact PSD array. The monomer structures are shown in Figure 2b: polyethylene glycol monophenyl ester acrylate (PEGPEA), di(ethylene glycol) ethyl ether acrylate (DEGEEA), hydroxyethyl methylacrylate (HEMA), poly(ethylene glycol) diacrylate (PEGDA), poly(ethylene glycol) dimethacrylate (PEGDMA), and hydroxyethyl acrylate (HEAA).

**FIGURE 2:**
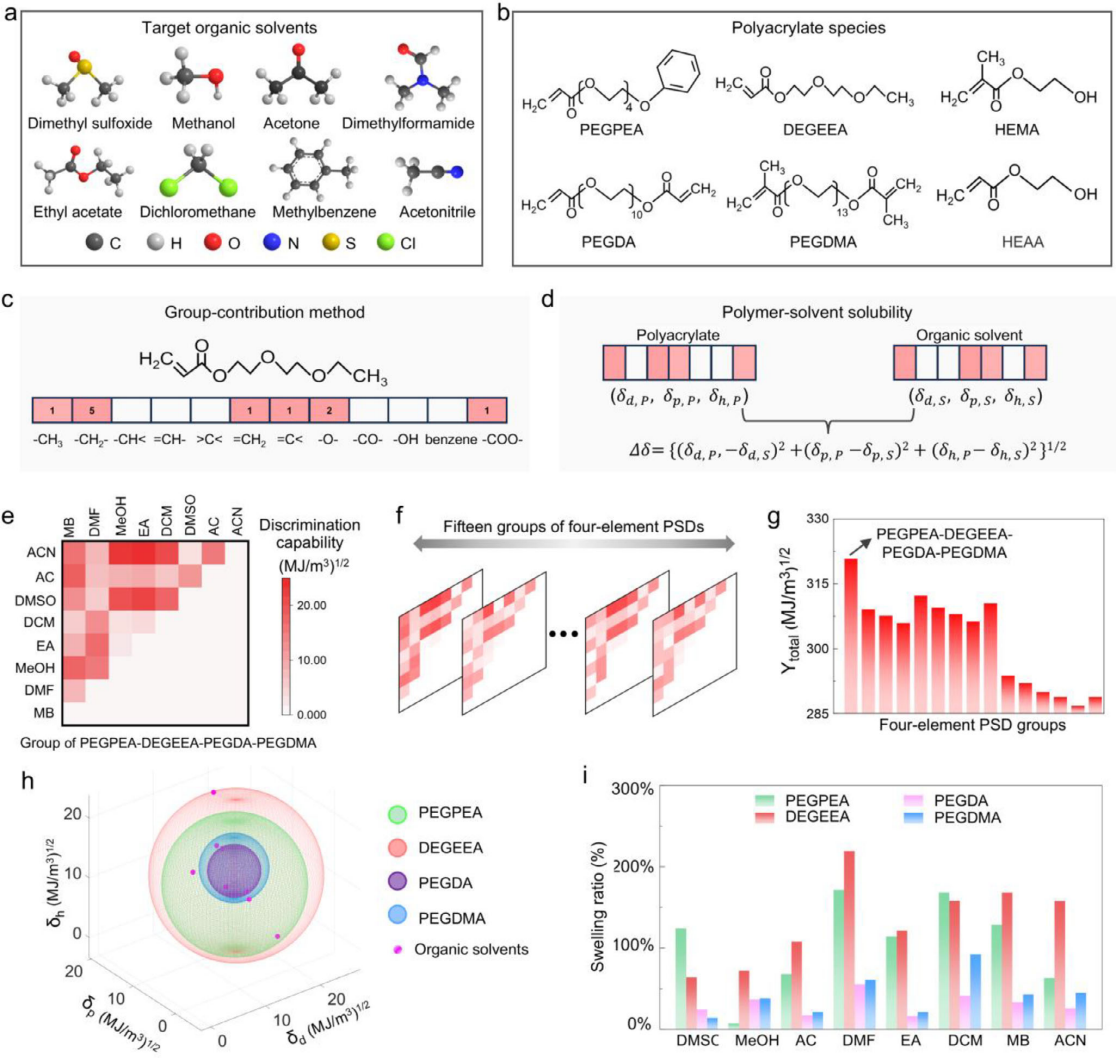
HSP‐guided chemical diversity of PSDs for maximized swelling cross‐reactivity. (a) Target organic solvent structures. (b) Polyacrylate monomer structures. (c) The group‐contribution method example (DEGEEA). (d) Determination of HSP difference to assess polymer‐solvent solubility. (e) Discrimination capability heatmap for the PEGPEA‐DEGEEA‐PEGDA‐PEGDMA array. (f) Discrimination capability heatmaps for all fifteen possible four‐element arrays. (g) *Y_total_* of the fifteen four‐element PSD groups. (h) 3D representation of polyacrylate HSP spheres and solvent HSP locations. (i) Experimental swelling ratios of PSDs in different solvents.

The HSPs (*δ_d_*, *δ_p_*, and *δ_h_* for the dispersion, polar, and hydrogen‐bonding interactions) of solvents and polyacrylates were calculated using a group‐contribution method of Hoftyzer‐Van Krevele with the Hanson Solubility Parameter in Practice (HSPiP) software package (Note S2). The solutes and solvents, taking DEGEEA as an example (Figure 2c), can be broken down into a combination of the numbers of constituent functional groups. The total solubility parameter *δ* is calculated in Equation (1):

(1) $$\delta =\sqrt{\delta_{d}^{2}+\delta_{p}^{2}+\delta_{h}^{2}}\;$$

Based on the similarity‐intermiscibility principle, polymer solubility in a solvent is determined by the HSP difference Δδ as expressed in Equation (2) [36]:

(2) $$\Delta \delta =\sqrt{{\left(\delta_{d,\;P}-\delta_{d,\;S}\right)}^{2}+{\left(\delta_{p,\;P}-\delta_{p,\;S}\right)}^{2}+{\left(\delta_{h,\;P}-\delta_{h,\;S}\right)}^{2}}\;$$

where subscripts *P* and *S* denote polymer and solvent, respectively. A small Δδ indicates good solubility and favorable polymer swelling (Figure 2d).

To select four polyacrylate species for a four‐element PSD array with maximized overall swelling cross‐reactivity, Δδ between polyacrylate *M* (polyacrylate species numbered from *A* to *F*) and solvent *n* (solvents numbered from one to eight) is denoted as *M_n_*. As a result, for a PSD array composed of four polyacrylate species (e.g., *A*, *B*, *C*, and *D*), the discrimination capability between solvents *x* and *y* is evaluated using *Y*_*x*,*y*_:

(3) $$\begin{array}{c} Y_{x,y}=\sqrt{{\left(A_{x}-A_{y}\right)}^{2}+{\left(B_{x}-B_{y}\right)}^{2}+{\left(C_{x}-C_{y}\right)}^{2}+{\left(D_{x}-D_{y}\right)}^{2}} \end{array}$$

The *Y*_*x*,*y*_ for all solvent pairs can be visualized in a heatmap matrix. Figure 2e shows the heatmap for the PEGPEA‐DEGEEA‐PEGDA‐PEGDMA array, indicating varying discrimination capability between different solvent pairs. Heatmaps of all fifteen possible four‐element combinations from the six polyacrylates are compared in Figure 2f. From these, the overall discrimination power *Y_total_* of a given array is assessed by the summing all the 28 *Y*_*x*,*y*_ (combinations of $C_{8}^{2}$). Figure 2g lists *Y_total_* for possible four‐element groups (detailed values in Table S3), ranging from 288 to 320 (MJ/m^3^)^1/2^. The PEGPEA‐DEGEEA‐PEGDA‐PEGDMA group exhibits the highest *Y_total_* value, indicating optimal chemical diversity for maximized cross‐reactivity.

For visual estimation, HSP spheres of the polyacrylates and the HSP locations of the target solvents were plotted in HSP space (Figure 2h; Note S2). Solvents inside a sphere indicate good solubility for the polyacrylate, while those outside suggest poor solubility. The eight solvents are evenly distributed from inner to outer HSP spheres of differing radii, suggesting gradually changing swelling capacity toward these solvents. We experimentally determined equilibrium swelling ratios of PSDs in different solvents (Section 4). The PSD array shows distinct swelling ratio combinations across solvents (Figure 2i), experimentally verifying the optimized chemical diversity for enhanced cross‐reactivity. The highest *Y_total_* group (denoted as P1 to P4) was used to construct the BiPC for subsequent performance testing.

### 2.3 Fast, Reversible, and Thermally Stable Sensing Performance

The color‐change capture setup is schematically shown in Figure 3a, involving a light‐shield box equipped with a white light‐emitting diode to cooperatively avoid external interference. The BiPC has a small form factor (50 × 35 mm^2^), and requires only 1.5 mL of liquid for sensing. The optimal subject distance *H* was adjusted to 20 cm; shorter distances led to an insufficient irradiation area to cover all PSDs, while longer distances provided inadequate brightness for clear color capture (Figure 3b). Preliminary observations showed the P3 PSD in DMF had the slowest color response, defining the minimum required detection time. To quantify the detection time, the color change process was video‐recorded, and the time‐evolved Euclidean distance in RGB space from the initial state was calculated (Figure 3c). Detection time increased with PSD thickness; a 50‐µm‐thick PSD showed a rapid color change of around 30s in DMF. Thus, a thickness of 50 µm was chosen for the BiPC PSDs. We emphasize that the HSP framework is employed to predict equilibrium swelling affinities, which determine the final photonic bandgap shift and hence the colorimetric pattern. The response time to reach equilibrium is governed by solvent diffusion kinetics, which depends on factors such as cross‐link density, polymer segmental mobility, and solvent molecular size. The 30‐s response time achieved with the 50‐µm‐thick PSDs indicates rapid solvent penetration, which is desirable for practical applications.

**FIGURE 3:**
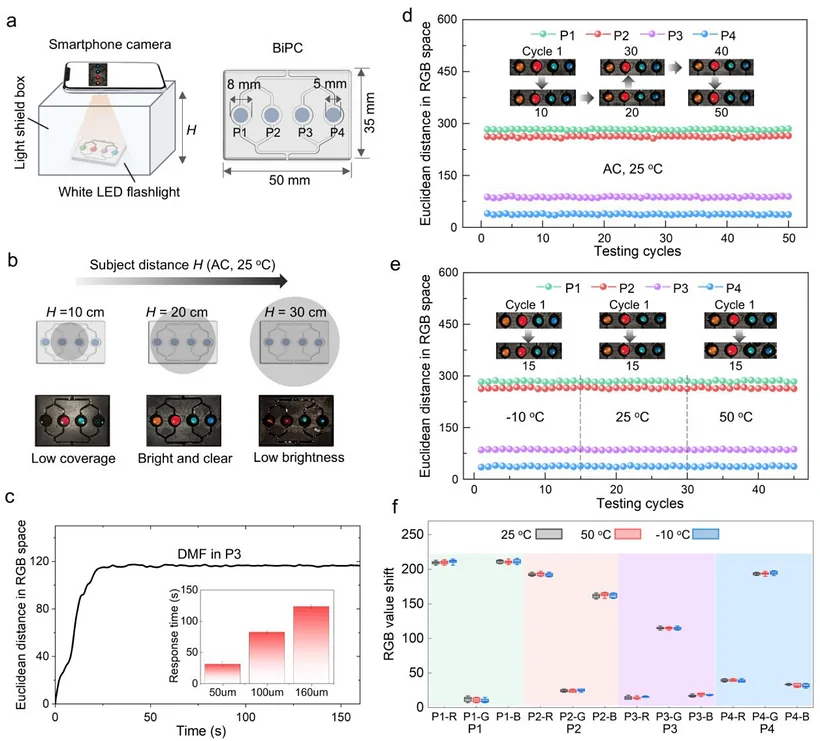
Fast, reversible, and thermally robust color response. (a) Schematic of the color‐capture setup and BiPC dimensions. (b) Optimization of subject distance for clear color capturing. (c) Time‐evolved color variation of P3 PSD in DMF in terms of Euclidean distance in RGB color space. Inset: response times of P3 PSD in DMF at different PSD thicknesses. (d) Color responses of BiPC to acetone over 50 cycles at 25°C. Insets: digital photos of color responses for increasing cycles. (e) Color responses of acetone in terms of Euclidean distance over 15 cycles at −10°C, 25°C, and 50°C. Insets: digital photos of color responses for increasing cycles at varied temperatures. (f) Averaged RGB value shifts of each color channel extracted from temperature‐varied cyclic tests.

Reversibility and thermal stability were evaluated using acetone as the test solvent (details in Section 4). Acetone was repeatedly added to the microfluidic channels at room temperature, and Euclidean distances of the four PSDs from the initial state were recorded (Figure S7). Figure 3d shows consistent color changes over 50 cycles, with constant Euclidean distances and negligible variation. Cyclic tests were also conducted at varying temperatures of −10°C, 25°C, and 50°C. Figure 3e shows almost unchanged colors over 15 cycles at each temperature. The averaged RGB value shifts for each color channel are shown in Figure 3f, demonstrating consistent RGB values across the temperature range to verify superb thermal stability in colorimetric sensing. The thermal stability tests demonstrate that the structural integrity and photonic properties remain intact across the temperature range of −10°C to 50°C. The small variations in RGB values across temperatures (standard deviations less than 5 units for each channel) indicate that the BiPC can operate reliably under typical environmental temperature fluctuations without requiring recalibration. The temperature dependence of solvent viscosity affects the kinetics but not the equilibrium color patterns, consistent with the thermodynamic stability of the sensor elements.

### 2.4 Single‐Solvent Recognition

Recognition of individual organic solvents was first investigated. Upon solvent addition to the BiPC, the initially bluish PSDs changed into different color patterns at swelling equilibrium (Figure 4a). These color patterns were transferred to colorimetric cards composed of four regenerated pure colors by statistical calculation of the averaged RGB values of the PSDs (Figure 4b, details in Section 4). The eight colorimetric cards displayed distinct patterns, allowing easy recognition of the individual solvents via intuitive color matching. The optimized cross‐reactivity owing to the HSP‐guided chemical diversity was substantiated by the varying Euclidean distances in RGB space of each PSD upon exposure to different solvents, as seen in Figure 4c. To develop a more quantitative recognition scheme, RGB value shifts of each PSD were extracted, exemplified by the heatmap matrix of DMSO in Figure 4d. These heatmaps were further transformed into radar patterns describing each color channel variation of the four‐element array. Figure 4e displays the RGB radar fingerprints for individual solvents, providing a multidimensional differentiation approach complementary to the colorimetric cards.

**FIGURE 4:**
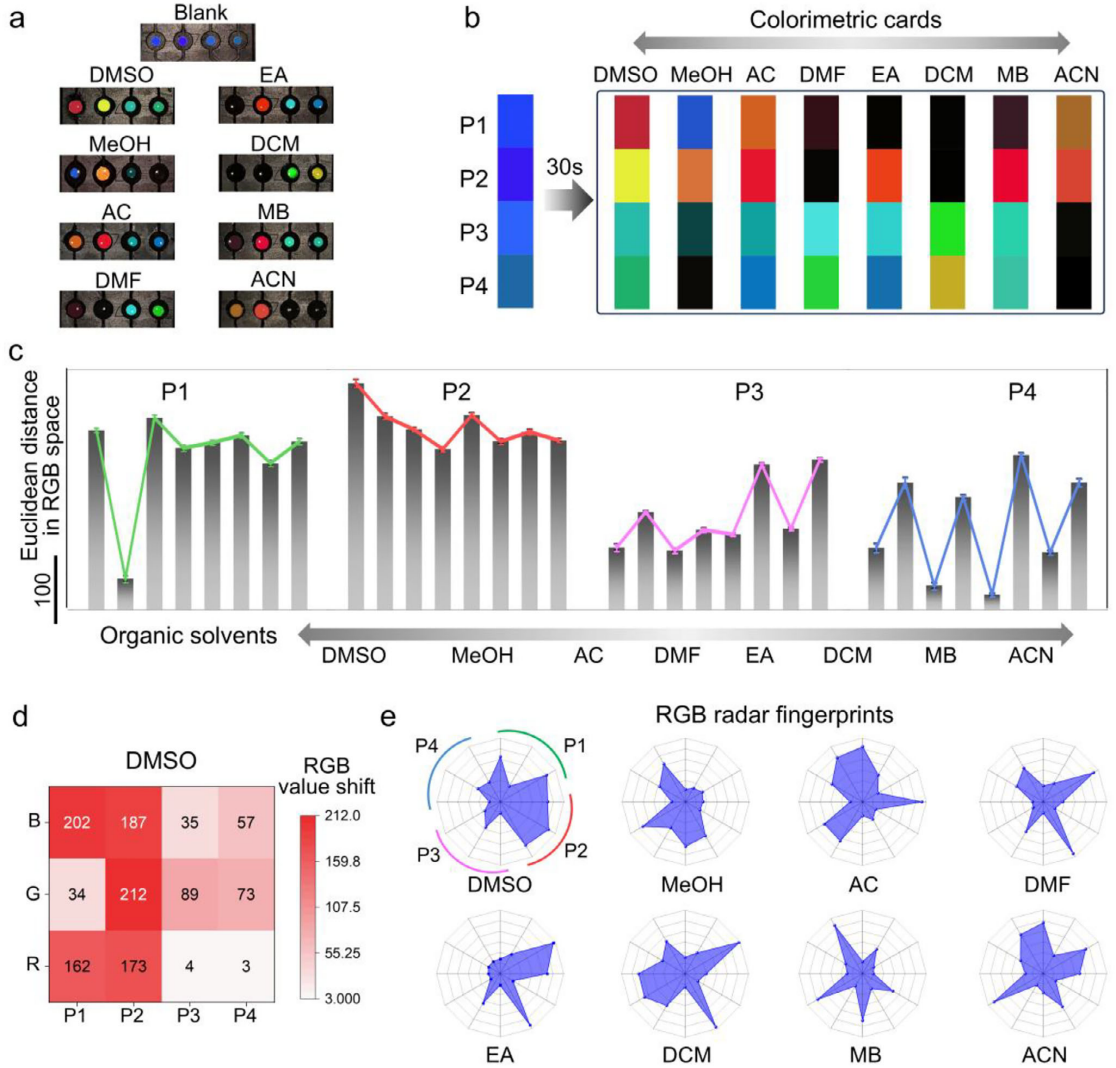
Single‐solvent recognition via intuitive pattern match. (a) Digital photos of the color patterns at blank and in response to individual organic solvents. (b) Colorimetric cards of the eight solvents. (c) Euclidean distances in RGB space for each PSD toward different solvents. (d) Heatmap matrix of RGB value shifts for DMSO. (e) RGB radar fingerprints for different solvents.

### 2.5 Identification of Component Concentration, Homologues, Isomers, and Liquors

Identifying component concentration in liquid mixtures remains challenging for existing quick sensing techniques. To examine quantification ability, acetone in ethanol with 10 wt.% stepwise concentration increases was used. Figure 5a shows the concentration‐evolved color responses. As acetone concentration increased from 10 to 100 wt.%, P1 PSD changed from sky blue to green and to orange, P2 from orange to red‐orange and to deep red, P3 from light green to blue green, and P4 from dark green to dark cyan and to navy blue. RGB values at equilibrium were plotted versus acetone concentrations in Figure 5b, showing remarkable concentration‐related fluctuations correlating with discernible color changes. Corresponding RGB radar fingerprints in Figure 5c displayed distinct radar patterns for differentiation. It should be noted that the performance metric reported here is classification accuracy rather than a conventional limit of detection (LOD). As a pattern‐recognition‐based sensor array, the BiPC is designed to generate distinctive response patterns for different liquids rather than to detect a specific analyte with high sensitivity. The concentration‐dependent color changes demonstrate the system's ability to differentiate liquids across a wide concentration range. The practical sensitivity threshold is determined by the smallest concentration step that yields statistically separable patterns, which in the current setup is 10 wt.% for acetone‐ethanol mixtures.

**FIGURE 5:**
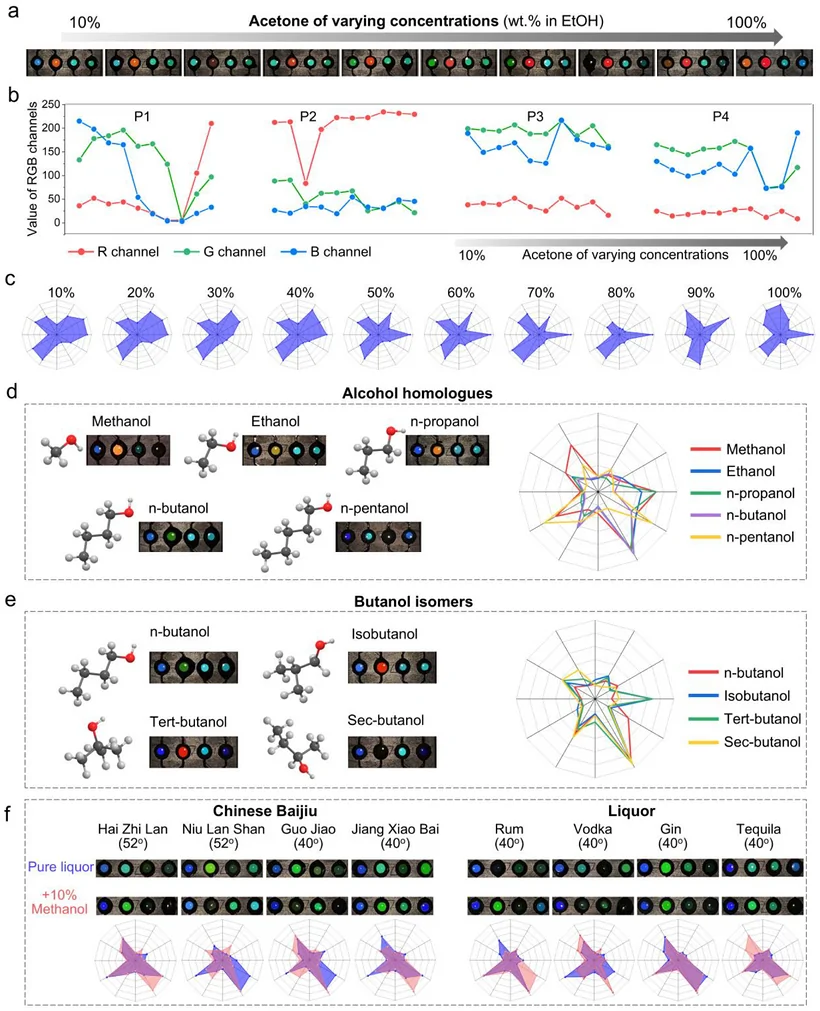
Identification of solvent concentrations, homologues, isomers and liquors. (a) Digital photos of the concentration‐evolved color responses of acetone in ethanol. (b) RGB values of individual PSDs versus acetone concentrations from 10 to 100 wt.%. (c) RGB radar fingerprints of different acetone concentrations. (d) Digital photos of color responses for alcohol homologues and the corresponding RGB radar fingerprints. (e) Digital photos of color responses for butanol isomers and the corresponding RGB radar fingerprints. (f) Digital photos of color responses of Chinese Baijiu (brands of Hai Zhi Lan, Niu Lan Shan, Guo Jiao, and Jiang Xiao Bai), liquors (rum, vodka, gin and tequila), and samples with 10 vol.% methanol added. RGB radar fingerprints show differences between pure and methanol‐contaminated liquors.

Beyond concentrations, the BiPC can identify homologues and isomers with similar structures. Alcohol homologues (methanol, ethanol, n‐propanol, n‐butanol, n‐pentanol) with very similar chemical structures and refractive indices (1.329, 1.361, 1.386, 1.399, and 1.410, respectively) produced markedly different color patterns, verified by the RGB radar fingerprints plotted in the same chart for comparison (Figure 5d). Swelling ratio deviations of alcohol homologues may arise from polarity and molecular size variations. Butanol isomers (n‐butanol, isobutanol, tert‐butanol, sec‐butanol) with nearly identical refractive indices (1.399, 1.396, 1.384, 1.397) also revealed distinctive color patterns and RGB radar fingerprints in Figure 5e, likely due to molecular conformation and hydrogen‐bond strength differences.

To demonstrate practicality as a facile and versatile tool for real‐life applications, the BiPC was used to identify different liquors (Figure S8) and methanol‐poisoned liquors. Figure 5f shows that four popular Chinese Baijiu brands revealed discernible color pattern discrepancies despite the same levels of alcohol by volume (ABV) at 52% and 40%. Four liquors (rum, vodka, gin, and tequila) with the same ABV level of 40% also displayed apparent color pattern differences. Methanol is a typical toxic and fatal component found in homebrewed liquors or illegal alcoholic beverages. Adding 10 vol.% of methanol to these Chinese Baijiu and liquors produced clear color responses compared to the initial color patterns of pure liquors. The differences in color patterns and RGB radar fingerprints between the original and laced liquors demonstrate potential for rapid screening of methanol contamination. Control experiments comparing the addition of 10 vol.% water versus 10 vol.% methanol to the same liquor showed clearly distinct colorimetric patterns (Figure S9), confirming that the BiPC can differentiate methanol adulteration from simple dilution.

### 2.6 AI‐Assisted On‐Site Recognition With the BiPC System

While the colorimetric cards and RGB radar fingerprints approaches are straightforward and cost‐effective, manual pattern matching is susceptible to subjective variations in color perception, particularly when handling large sample sets or operating under suboptimal lighting conditions. To address this, we implemented a deep learning algorithm to classify solvents based on the color response patterns generated by the BiPC. To validate the effectiveness, organic solvents mixed with ethanol in ten graded concentrations (10 wt.% intervals) were selected as target analytes (color response patterns in Figure S10). In this AI‐assisted recognition model, each concentration level was treated as a distinct category, resulting in a total of 80 recognizable liquid classes. This resulted in 80 distinct liquid samples (8 solvents in 10 concentrations) with unique colorimetric patterns. The YOLOv11 model was trained to classify these 80 pattern classes, achieving an overall accuracy of 97.1%. We note that while the concentration levels of each solvent produce distinct patterns, the 80 classes represent varying compositions rather than 80 chemically distinct species.

The integration of the BiPC with this algorithm constitutes a biomimetic gustatory system. Figure 6a,b illustrate the workflow block diagram and operational interfaces: (i) a smartphone camera captures the color pattern of the BiPC inside a light‐shield box; (ii) the user invokes a cloud application programming interface (API) to submit the image for cloud‐based computation; and (iii) classification results along with confidence scores are returned to the smartphone interface. For real‐time detection and classification, a YOLOv11 deep learning model was deployed on the cloud platform (Figure 6c and Note S3 for details). This cloud‐API implementation offers greater computational efficiency and broader compatibility compared to hosting the model locally on mobile devices. Using the optimized four‐element PSD array within the BiPC, the confusion matrix in Figure 6d compares true versus predicted liquid identities, demonstrating high prediction accuracy. Across the entire dataset (comprising 421 samples spanning 80 liquid classes), the model achieved an overall prediction accuracy of 97.1%. After 100 training epochs, the training loss (validation loss) dropped from initial values of 3.7 (2.8) to 0.36 (0.24), confirming the robustness and reliability of the YOLOv11 model in classifying colorimetric responses (Figure 6e).

**FIGURE 6:**
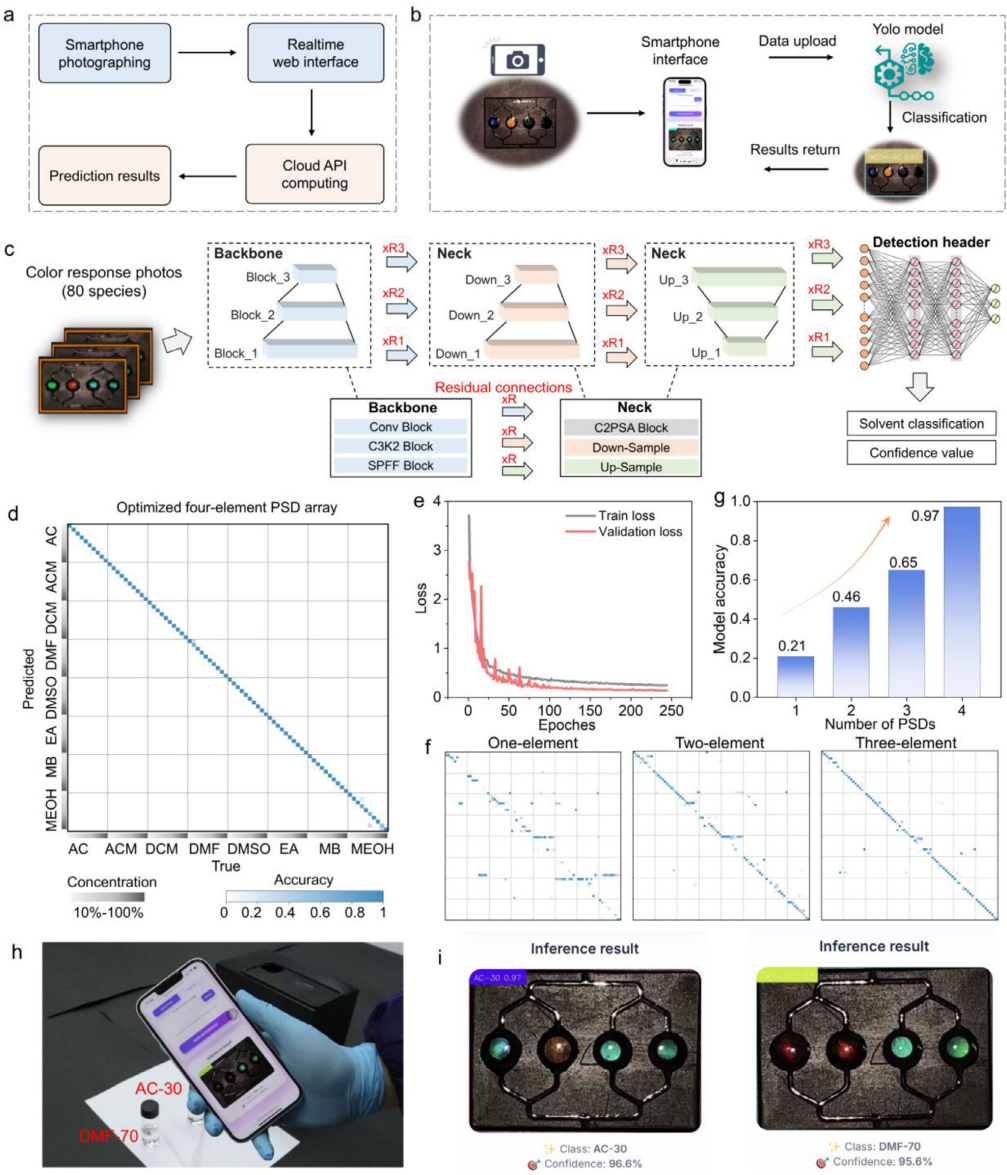
AI‐assisted on‐site recognition. (a) Block diagram and (b) operational interfaces of the AI‐assisted recognition process. (c) Schematic of the convolutional neural network (CNN) for solvent classification. (d) Confusion matrix of identification results using the optimized four‐element PSD array for recognizing 80 solvent classes (ten graded concentrations of eight organic solvents, concentration interval of 10 wt.%). (e) Training and validation curves across epochs when recognizing 80 solvent classes. (f) Comparison of classification confusion matrices using one‐, two‐, and three‐element sensor arrays when recognizing the same set of 80 solvent classes. (g) Model accuracy as a function of the number of PSDs in the BiPC. (h) Simulated on‐site identification of two solvent samples: AC of 30 wt.% in ethanol and DMF of 70 wt.% in ethanol. (i) Identification results displayed on the smartphone interface.

Further experiments were conducted to examine the relationship between the scale of the PSD sensor array and the corresponding identification accuracy. To ensure a fair comparison, the deep learning model and size of the dataset remained identical to those used for the four‐element PSD array. Classification confusion matrices for three additional BiPCs comprising different numbers of sensing elements (specifically, arrays of PEGPEA, PEGPEA‐DEGEEA, and PEGPEA‐DEGEEA‐PEGDA) are presented in Figure 6f. As summarized in Figure 6g, the model accuracy increased markedly from 20.7% (with one PSD) to 97.1% (with four PSDs), confirming the beneficial role of array scale in enhancing identification performance. For more challenging sensing tasks (e.g., discriminating multi‐component liquid mixtures, detecting finer concentration differences), employing a larger PSD sensor array represents a reliable strategy for achieving high accuracy. The proposed HSP‐guided chemical diversity framework offers a pathway to downsizing the sensor array, thereby mitigating the associated challenges of increased fabrication complexity, larger sample requirements, visual interpretation errors, and data redundancy. To demonstrate on‐site, real‐time recognition, AC (30 wt.% in ethanol) and DMF (70 wt.% in ethanol) were selected as test samples (Figure 6h and Movie S1). The results shown in Figure 6i substantiate the excellent identification capability of the BiPC system. The end‐user operation involves three simple steps: (i) pipetting 1.5 mL of the liquid sample into the chip, (ii) waiting 30 s for the color response to stabilize, and (iii) capturing an image using a smartphone camera through a dedicated light‐shield box. The entire procedure is performed within a compact, portable setup that can be deployed in field settings without requiring specialized equipment or training. By integrating cloud computing via a smartphone, the BiPC‐based platform offers a fast, low‐cost, highly accurate, and field‐deployable sensing technology suitable for various applications such as chemical hazard inspection and chemical production monitoring.

## 3 Conclusion

This study introduces a biomimetic photonic‐gustatory chip designed for rapid, accurate, and on‐site identification of organic liquids. By strategically employing an HSP framework, a compact polyacrylate‐based sensor array with maximized cross‐reactivity was developed. The resulting BiPC effectively differentiates a broad spectrum of analytes, including pure solvents, mixtures across varying concentrations, homologues, isomers, and commercial alcoholic beverages. Furthermore, integration with a smartphone application and a deep learning algorithm enables automated, cloud‐assisted pattern recognition, achieving high accuracy (97.1% across 80 liquid classes) within 30 s. While the current BiPC design employs a 1.5 mL sample volume for reliable operation, the system architecture is readily amenable to miniaturization. Through scaling of the microcavity dimensions, reduction of PSD thickness, and adoption of flow‐through or droplet‐based configurations, sample consumption could be reduced to the microliter range without compromising sensing performance. Such optimizations would further enhance the utility of the BiPC for precious or hazardous liquid analysis. For open‐world deployment, the classification model can be coupled with confidence‐thresholding or out‐of‐distribution detection, allowing samples that fall outside the trained categories to be flagged as ‘Unknown’ rather than being forced into a false‐positive classification. This work highlights the considerable potential for portable, low‐cost chemical sensing for applications in hazard inspection, quality control, and food safety. The HSP‐guided design principle is also readily adaptable to a wider range of liquid systems and environmental conditions, thereby supporting its use in complex real‐world sensing scenarios.

## 4 Methods

### 4.1 Materials and Characterization

Ethanol (EtOH, AR, 99%), n‐Butanol (AR, 99%), toluene (MB, ≥ 99.5%), methanol (MeOH, ≥ 99.5%), acetone (AC, ≥ 99.5%) were purchased from Shanghai Lingfeng Chemical Reagent Co., Ltd., Tetraethyl orthosilicate (TEOS, AR, 99%), isopropanol (AR, 99%), sec‐butanol (AR, 99%), ammonia (NH3‐H2O, AR), acetonitrile (ACN, ≥ 99%), dimethyl sulfoxide (DMSO, ≥ 99.5%), ethyl acetate (EA, ≥ 99.5%), and N,N‐dimethylformamide (DMF, ≥ 99.5%) were purchased from Sinopharm Chemical Reagent Co., Ltd., Polyethylene glycol phenyl ether acrylic acid (PEGPEA, MW 324) and diethylene glycol ethyl ether acrylic acid (DEGEEA, MW188) were purchased from Sigma–Aldrich Trading Co., Ltd., Polyethylene glycol diacrylate (PEGDA, MW575), polyethylene glycol diacrylate (PEGDA, MW700), n‐propanol (AR, 99%), tert‐butanol (AR, 99%), dichloromethane (DCM, ≥ 99.9%) were purchased from Shanghai Macklin Biochemical Co., Ltd., 2‐Hydroxy‐2‐methylpropiophenone (Irgacure1173, MW164) and n‐pentanol (AR, 99%) was purchased from Shanghai Taitan Technology Co., Ltd. Niu Lan Shan (52° ABV, Niulanshan Distillery of Beijing Shunxin Agricultural Co., Ltd.), Hai Zhi Lan (52° ABV, Jiangsu Yanghe Distillery Co., Ltd.), Guo Jiao (52° ABV, Luzhoulaojiao Co. Ltd.), Jiang Xiao Bai (40° ABV, Chongqing JiangxiaoBai Co. Ltd.), Vodka (40° ABV, Pernod Ricard (China)), Rum (40° ABV, Bacardi Spirits Trading Company), Gin (40° ABV, Pernod Ricard (China)) Tequila (40° ABV, chinabev(Ningbo) Trading Co., Ltd.).

### 4.2 Fabrication of BiPC

A microchip was prepared using a polytetrafluoroethylene (PTFE) substrate with a thickness of 5 mm via computer numerical control (CNC) machining technology. The microchip contains four circular microcavities (diameter of 8 mm, depth of 1.5 mm) distributed in a linear array, and each microcavity is interconnected by 1.0 mm diameter microfluidic channels (Figure S1). The prepared PSDs are placed in the microcavities to form a multi‐element sensory array.

### 4.3 Fabrication of PSDs

#### 4.3.1 Monodisperse Silica Nanospheres

Silica nanospheres were synthesized by a modified Stöber's method (Figure S2a) [37, 38, 39]. Ethyl orthosilicate hydrolyzes and condenses under an alkaline environment (ammonia) to form monodisperse silica nanospheres. Anhydrous ethanol, ammonia, and distilled water in the ratio of 100:4:7 were taken and mixed well in a three‐necked beaker, then ethyl orthosilicate was added dropwise by titration, and the reaction was carried out for 8 h to obtain silica nanospheres with a diameter of 128 nm.

#### 4.3.2 Light‐Curable Colloidal Dispersions

A suspension of monodisperse silica nanospheres was purified by centrifugation and ultrasound treatment. The purified silica nanospheres were homogeneously dispersed in ethanol, and the suspension was mixed with an acrylate matrix containing 1 w/w % 2‐hydroxy‐2‐methyl‐1‐phenyl‐1‐propane photoinitiator. The amount of acrylate was determined by the weight of the silica in the suspension to obtain the target volume fractions of the silica nanospheres. The light‐curable colloidal dispersion was prepared by selective evaporation of anhydrous ethanol in an oven at 80°C for 12 h.

#### 4.3.3 Elastic PSD Membrane

The light‐curing dispersion was introduced into the gap between two glass slides via capillary forces using a spacer layer of polyimide tape (Kapton) (Figure S2b). After around 5 min, the structural color appeared. The thickness of the photonic crystal film was determined by the polyimide tape, and the dispersions were irradiated with a UV‐curing chamber (Cure 100N, Lichtzen) with an intensity of 100% for 20 s. The photonic crystal film was cut into disk‐shaped membranes with a diameter of 5 mm.

### 4.4 PSD Array Color Data Extraction

The optical photos of the PSD arrays collected with the smart device were imported into Adobe Photoshop, and the color extraction tool was used to extract the RGB values of the array units by using a 5 × 5 extraction range (25 pixel points) for the more uniformly colored parts of the sensor array units. This process was repeated three times for three independent experiments, and finally, the extracted RGB values were averaged. The actual classification results in AI‐assisted prediction were obtained using the YOLOv11 deep learning model, which processes the entire image (640 × 640 pixels) through automated feature extraction rather than manual RGB averaging.

### 4.5 Reversibility and Thermostability Test

Under a given test temperature condition, after adding the organic solvent dropwise, record the color information once the film color reaches equilibrium. Subsequently, remove the organic solvent from the film via evaporation, restoring it to its initial state before extracting the color information once more. Repeat the aforementioned experimental steps.

### 4.6 Equilibrium Swelling Experiment

Use an electronic balance to weigh the dried photonic crystal sensor film unit sample and record its initial mass m_0_. Make sure that the sample is not affected by moisture in the air; choose the organic solvent to be measured first, take ethanol as an example, use a pipette gun to take 3 mL of ethanol and inject it into the sample box, take a piece of the photonic crystal film with a radius of 2.5 mm and allow the ethanol liquid to completely submerge the film; observe the swelling process of the photonic crystal unit sample within a certain period of time. The swelling experiment can be carried out at room temperature, and the mass of the sample can be recorded at regular intervals (every 6 h). During the swelling process, solvent molecules penetrate into the film, causing it to expand in volume. After 72 h of swelling, the swollen polymer sample is removed using filter paper to remove excess solvent from the surface. Make sure there is no residual solution left in the sample. The swollen polymer samples were accurately weighed using an electronic balance; the mass m_t_ (mass after swelling) was recorded, and the equilibrium swelling rate was calculated using the swelling formula. Three independent sets of equilibrium swelling experiments were performed and averaged to ensure the reliability of the results.

### 4.7 BiPC Data Acquisition and Intelligent Analysis Pipeline

The BiPC is placed in a dark box with a length, width, and height of 20 cm, and the lighting equipment that comes with the smartphone is used to provide vertical light; 1.5 mL of the solution is taken and added to the microfluidic device. After waiting for around 30 s, take a photo of the BiPC and upload it to the cloud via a web interface. The trained YOLOv11 model is used to automatically identify the solution and return the results to the smartphone application interface.

## Author Contributions

Conceptualization: J.Z., Y.C., and R.W. Methodology: J.Z., H.O., L.Z., Y.C., and R.W. Investigation: J.Z., X.L., and Y.C. Data curation: J.Z. and H.O. Validation: J.Z., H.O., and Y.C. Formal analysis: J.Z., H.O., Y.C., and R.W. Resources: J.Z., H.O., J.S., and R.W. Visualization: J.Z., H.O., Y.C., and R.W. Software: L.Z., G.Z., N.X., and X.M. Funding acquisition: R.W., Y.C., and J.S. Project administration: R.W. and Y.C. Supervision: R.W., Y.C., and L.Z. Writing – original draft: J.Z. and Y.C. Writing – review & editing: R.W., Y.C., L.Z., H.O., and J.Z.

## Funding

This work was financially supported by the National Key Research and Development Program of China (Grant No. 2024YFB3814100), the National Natural Science Foundation of China (62471459, U24A20228, and 62301540), the Natural Science Foundation of Shaanxi Province (2024JCJCQN‐66), the Chinese Academy of Sciences Project for Young Scientists in Basic Research (Grant No. YSBR‐109), the Joint Project “ActFiber‐FFG/CAS: 931688/030GJHZ2025029MI” managed by the Austrian Research Promotion Agency (FFG) and the Chinese Academy of Sciences (CAS), the Shanghai International Science and Technology Cooperation Project (23520711000), and the Shanghai Rising Star Program (24QA2710600) from Science and Technology Commission of Shanghai Municipality.

## Conflicts of Interest

The authors declare no conflicts of interest.

## Supporting information




**Supporting File 1**: advs78158‐sup‐0001‐SuppMat.docx.

**Supporting File 2**: advs78158‐sup‐0002‐MovieS1.mp4.

## Data Availability

The data that support the findings of this study are available from the corresponding author upon reasonable request.
