# Supplementary material for: A Biomimetic Solvatochromic Chip With Optimized Chemical Diversity for On‐Site Liquid Identification
Source: Adv Sci (Weinh). 2026 Oct 8:e78158. Online ahead of print. doi: 10.1002/advs.78158 (PMC13650038; doi:10.1002/advs.78158)
Supplement: Supplementary file 1 [file ADVS-9999-e78158-s002.docx]

Supplementary Materials for

**A biomimetic solvatochromic chip with optimized chemical diversity for on-site liquid identification**

Jintao Zhang^1†^, Hao Ouyang^1,2†^, Yin Cheng^1^^†,^^*^, Nuoye Xiong^3†^, Xunchi Ma^3^, Xueling Li^1^, Guangming Zhu^3^, Liang Zhang^3*^, Jing Sun^1^, Ranran Wang^1,4*^

1. The State Key Laboratory of High Performance Ceramics, Shanghai Institute of Ceramics, Chinese Academy of Sciences, Shanghai 200050, China
2. Center of Materials Science and Optoelectronics Engineering, University of Chinese Academy of Sciences, Beijing 100049, China
3. School of Computer Science and Technology, Xidian University, Xi’an 710071, China
4. School of Chemistry and Materials Science, Hangzhou Institute for Advanced Study, University of Chinese Academy of Sciences, Hangzhou 310024, China

*Corresponding author. Email: Yin Cheng, [chengyin@mail.sic.ac.cn](mailto:chengyin@mail.sic.ac.cn); Liang Zhang, [liangzhang@xidian.edu.cn](mailto:liangzhang@xidian.edu.cn); Ranran Wang, [wangranran@mail.sic.ac.cn](mailto:wangranran@mail.sic.ac.cn)

**This PDF file includes:**

Supplementary Note 1 to 3

Tables S1 to S3

Figs. S1 to S10

**Other Supplementary Materials for this manuscript include the following:**

Movies S1. On-site solvent recognition using BiPC-based platform

Supplementary Notes

Supplementary Note 1: Solvent-induced swelling in PSD color response

During the response of PSD to solvents, the color change primarily stems from two mechanisms: firstly, the swelling effect of the solvent causes a volume change in the polymer matrix, thereby altering the lattice spacing *d*; secondly, the absorption effect of the solvent modifies the effective refractive index *n_eff_* of the PSD.

To determine the dominant role of PSD in color changes across different solvents, this study selected three sets of organic solvents with similar refractive indices for experimentation: acetone and ethanol (refractive index 1.36), dimethylformamide and ethylene glycol (refractive index 1.43), and methanol and water (refractive index 1.33). To ensure experimental accuracy, measurements of RGB chromaticity changes (RGB distance) were conducted simultaneously across all four PSDs. The three sets of organic solvents with identical refractive indices exhibited differing degrees of color variation within the same polymeric photonic crystal system (Fig S6). This finding indicates that the swelling effect of organic solvents on the polymer substrate, rather than solvent absorption, dominates the color-changing process in PSDs.

Supplementary Note 2: Calculation and visualization of Hansen solubility parameters

The four PSD units (PEGPEA, DEGEAA, PEGDA, PEGDMA) of the array were subjected to dissolution experiments in eight solvent solvents (acetone, methanol, dimethylsulfoxide, dimethylformamide, acetonitrile, ethyl acetate, dichloromethane, and toluene), and the equilibrium dissolution rate of the array units in eight solvents was obtained through the dissolution experiments, and the data were used to judge the good or bad dissolution. The good swelling is indicated by "1", the bad swelling is indicated by "0", we use the HSPiP software to fill in the data. At the same time, the three Hansen solubility parameters (dispersion component, polarity component, and hydrogen bonding component) of the polymer were calculated using the Hansen solubility parameter calculation method, and they were imported into the genetic algorithm (GA) in the HSPiP for calibration, and the algorithm would calculate the interaction between the polymer and the organic solvent.

The R_0_ of the polymer (*i.e.* the radius of the solute Hansen sphere in Hansen space) is calculated by this method, and the center of the sphere represents the three components of the Hansen solubility sphere of the polymer, together plotting the Hansen Solubility Parameter Sphere (HSP sphere). If the HSP location of a certain solvent fall within the Hansen sphere of a polymer, *i.e.*, the spacing between the HSP of the polymer and the HSP of that solvent (Ra, RED < 1) (Principle 1), there is a good affinity between the polymer and the solvent, and vice versa, if the RED is > 1, there is a poor affinity between the two.

$$\begin{aligned} \text{Ra=}\left[ \text{4}\left( \text{δ}_{\text{d,p}}\text{-}\text{δ}_{\text{d,s}} \right)^{\text{2}}\text{+}\left( \text{δ}_{\text{p,p}}\text{-}\text{δ}_{\text{p,s}} \right)^{\text{2}}\text{+}\left( \text{δ}_{\text{h,p}}\text{-}\text{δ}_{\text{h,s}} \right)^{\text{2}} \right]^{\frac{\text{1}}{\text{2}}}\text{(}\text{Principle 1}\text{)} \end{aligned}$$

$$\text{RED=}\frac{\text{Ra}}{\text{Ro}}$$

Supplementary Note 3: Deep-learning model of YOLOv11 for detection and classification

We seamlessly integrated the YOLOv11 model into our solvent detection pipeline to conduct the classification process. Below we provide a concise abstract description and the relevant operator calculus. The data dimensions of the input raw image are shown as follows:

$$I_{\text{raw}}\in R^{H\times W\times\mathbb{3}},H=W=640$$

Since the tensor input of YOLOv11 is a square, the H and W here is 640 pixels, and the multiplier 3 denotes that the color space of the picture here is RGB. Then the raw data will go through a preprocessing process mainly to calibrate the overexposure caused by the flashlight on the mobile devices.

The YOLOv11 model handles the input data and performs forward propagation to predict the location and the classes of the solvent detection array. The whole process can be denoted as:

$${\mathcal{\mathcal{F}}}_{\text{YOLO v11}}: R^{H\times W\times\mathbb{3}} \to R^{S\times S\times\left( B\cdot\left( \mathbb{4+1+}C \right) \right)}$$

Where H and W represent the height and width of the input image, 3 denotes the RGB channels, S x S indicates the grid division, B is the number of bounding boxes predicted per grid, 4 corresponds to the bounding box parameters (x, y, w, h), 1 represents the prediction score, and C is the number of classes, which is 80.

The YOLOv11 model ${\mathcal{\mathcal{F}}}_{\text{YOLO v11}}$ has a backbone–neck–head architecture with Backbone producing multi-scale feature maps $F_{1},F_{2},F_{3}$, Neck performing upsample/downsample/concat based feature fusion, and the Detect Head emitting per-grid prediction vectors encoding box regressors, object and class logits for the solvent classes.

Prediction results need to be decoded and merged to transform the tensor into the output data format. It is processed by confidence thresholding $\tau$ followed by Non-Maximum Suppression (NMS):

$$\mathcal{D}_{\text{cand}}=\{ d\in\bigcup_{s} T_{s}\mid p_{\text{obj}}\left( d \right)>\tau_{\text{conf}} \}$$

$$\mathcal{Y=}\text{NMS}\left( \mathcal{D}_{\text{cand}};\tau_{\text{iou}} \right)$$

After the pipeline, the output result is as follows:

$$\mathcal{Y=}\{\hat{y_{i}}\}$$

$$\hat{y_{i}}=\left( b_{x,i},b_{y,i},b_{w,i},b_{h,i},p_{\text{obj},i},p_{\text{cs}i} \right)$$

where$\left( b_{x,i},b_{y,i} \right)$denote the center coordinates of the predicted bounding box, $\left( b_{w,i},b_{h,i} \right)$indicate its width and height, $p_{\text{obj},i}\in\left[ 0,1 \right]$corresponds to the estimated probability, and $p_{\text{cs}i}\in\left[ 0,1 \right]^{C}$represents the class probability vector over 80 solvent categories.

Table S1: Performance comparison with the existing solvent recognition mechanisms

| Recognition mechanism | Accessibility and Portability | Testing time | Identifying organic liquids(types) | Resistant to temperature interference | Ref. |
| --- | --- | --- | --- | --- | --- |
| BiPC | High (equipment: smartphone) | 25s | 80 | high | **our work** |
| Dynamic reflection spectrum | Low (equipment: ultraviolet–visible spectrometer) | 600s | 30 | low | [9] |
| Transparency-switching media | High (equipment: smartphone) | 30s | 19 | medium | [10] |
| Triboelectric-visual fusion | Medium (equipment: electrical signal acquisition module & industrial camera) | 1s | 13 | low | [11] |

**Table S2. Performance comparison with existing colorimetric sensors**

| Colorimetric sensors | Detection time | Detectable types per sensory element | Ref. |
| --- | --- | --- | --- |
| BiPC | 25 s | 80/4 | **our work** |
| Dye-based sensing array | 3 h | 8/12 | [16] |
| Solvatochromic sensor array | 5 min | 11/18 | [17] |
| Chemically responsive inks sensor array | 2 min | 14/36 | [18] |
| Solvato/vapochromic metal-organic polyhedron | 60 s | 8/6 | [19] |
| Cu/nucleotide MOFs sensor  array | 20 min | 36/3 | [20] |
| Vapochromic MOP aerogels | 60 min | 6/1 | [21] |
| Solvatochromic porous MOFs | 1 min | 7/1 | [22] |
| Smartphone-Based VOC Sensor | 30 min | 11/4 | [23] |
| PDAs film | 5 min | 15/6 | [24] |
| PDAs film | 10 s | 3/4 | [25] |
| PDAs film | 5 min | 20/4 | [26] |
| PEGDA Films | 1 min | 14/1 | [27] |
| CNC-based films | 1 min | 10/1 | [28] |
| Crystalline colloidal array | 10 min | 7/1 | [29] |
| CNC-based films | 1 min | 6/1 | [30] |
| Hollow-Sphere Photonic Crystals | 20 min | 7/1 | [31] |

Table S3. The Y_total_ of the 15 possible four-element PSD groups

| **combination** |  |  |  |  | **Y_total_** |
| --- | --- | --- | --- | --- | --- |
| **1** | **PEGPEA** | **DEGEAA** | **PEGDA** | **PEGDMA** | **320.77** |
| **2** | **PEGPEA** | **HEAA** | **PEGDA** | **PEGDMA** | **309.08** |
| **3** | **PEGPEA** | **DEGEAA** | **HEAA** | **PEGDMA** | **307.62** |
| **4** | **PEGPEA** | **DEGEAA** | **PEGDA** | **HEAA** | **305.96** |
| **5** | **HEAA** | **DEGEAA** | **PEGDA** | **PEGDMA** | **312.22** |
| **6** | **PEGPEA** | **HEMA** | **PEGDA** | **PEGDMA** | **309.43** |
| **7** | **PEGPEA** | **DEGEAA** | **HEMA** | **PEGDMA** | **307.99** |
| **8** | **PEGPEA** | **DEGEAA** | **PEGDA** | **HEMA** | **306.25** |
| **9** | **HEMA** | **DEGEAA** | **PEGDA** | **PEGDMA** | **310.49** |
| **10** | **HEMA** | **HEAA** | **PEGDA** | **PEGDMA** | **293.74** |
| **11** | **HEMA** | **DEGEAA** | **HEAA** | **PEGDMA** | **292.04** |
| **12** | **HEMA** | **DEGEAA** | **PEGDA** | **HEAA** | **289.94** |
| **13** | **PEGPEA** | **HEAA** | **HEMA** | **PEGDMA** | **288.79** |
| **14** | **PEGPEA** | **HEAA** | **PEGDA** | **HEMA** | **286.76** |
| **15** | **PEGPEA** | **DEGEAA** | **HEAA** | **HEMA** | **288.79** |

**Figure S1. Schematic of the BiPC dimensions and assembly of PSDs into the BiPC.**

**Figure S2. Schematic of the fabrication process of PSD.** (a) Monodisperse silica nanosphere synthesis. (b) Elastic photonic crystal PSD membrane fabrication. (c) Non-closed packed hexagonal lattices of silica nanospheres embedded in a solvent-free polyacrylate matrix, DEGEEA as an example.

**Figure S3. The relationship between the color of PSDs from four different polyacrylate species and the volume fraction of silica.**

**Figure S4. RGB values of each PSD color from three independent preparation batches.**

**Figure S5. The color of the PSDs stored at room temperature (RT, 25°C), low temperature (LT, -10 °C), and high temperature (HT, 80 °C) for 12 months, respectively.** (a) The RGB values; (b) The optical image.

**Figure S6. Color responses of each PSD in terms of Euclidean RGB distance corresponding to three pairs of organic solvents with similar refractive indices.**

**Figure S7. Euclidean RGB distance variation of each PSD at room temperature over 50 testing cycles.**

**Figure S8. Liquors of different brands for test, including four Chinese Baijiu brands and four base liquors.**

Figure S9. Figure S9. The optical photographs and corresponding RGB radar fingerprints for pure liquors (Chinese Baijiu of Niu Lan Shan and liquors of gin), liquors with 10% methanol added, and liquors with 10% water added.

**Figure S10. Color response patterns of eight organic solvents at different mix ratios with ethanol.**
